# Children’s use of egocentric reference frames in spatial language is related to their numerical magnitude understanding

**DOI:** 10.3389/fpsyg.2022.943191

**Published:** 2022-07-22

**Authors:** Nadja Lindner, Korbinian Moeller, Frauke Hildebrandt, Marcus Hasselhorn, Jan Lonnemann

**Affiliations:** ^1^Empirical Childhood Research, University of Potsdam, Potsdam, Germany; ^2^Centre for Mathematical Cognition, Loughborough University, Loughborough, United Kingdom; ^3^Leibniz-Institut fuer Wissensmedien, Tübingen, Germany; ^4^Department of Psychology, LEAD Graduate School and Research Network, Eberhard Karls University Tübingen, Tübingen, Germany; ^5^Center for Research on Individual Development and Adaptive Education of Children at Risk (IDeA), Frankfurt am Main, Germany; ^6^Department of Social and Educational Sciences, University of Applied Sciences, Potsdam, Germany; ^7^Department of Education and Human Development, DIPF | Leibniz Institute for Research and Information in Education, Frankfurt am Main, Germany

**Keywords:** spatial language, frames of reference, numerical development, mental number line, preschool children

## Abstract

Numerical magnitude information is assumed to be spatially represented in the form of a *mental number line* defined with respect to a body-centred, egocentric frame of reference. In this context, spatial language skills such as mastery of verbal descriptions of spatial position (e.g., in front of, behind, to the right/left) have been proposed to be relevant for grasping spatial relations between numerical magnitudes on the mental number line. We examined 4- to 5-year-old’s spatial language skills in tasks that allow responses in egocentric and allocentric frames of reference, as well as their relative understanding of numerical magnitude (assessed by a number word comparison task). In addition, we evaluated influences of children’s absolute understanding of numerical magnitude assessed by their number word comprehension (montring different numbers using their fingers) and of their knowledge on numerical sequences (determining predecessors and successors as well as identifying missing dice patterns of a series). Results indicated that when considering responses that corresponded to the egocentric perspective, children’s spatial language was associated significantly with their relative numerical magnitude understanding, even after controlling for covariates, such as children’s SES, mental rotation skills, and also absolute magnitude understanding or knowledge on numerical sequences. This suggests that the use of egocentric reference frames in spatial language may facilitate spatial representation of numbers along a mental number line and thus seem important for preschoolers’ relative understanding of numerical magnitude.

## Introduction

According to the Organisation for Economic Co-operation and Development (OECD) standards, academic skills such as mathematics proficiency are increasingly important in order to fully exercise citizenship rights (OECD, 2018). For instance, Ritchie and Bates (2013) found that mathematical skills at the age of seven significantly predicted socioeconomic status (SES) in adulthood. Thus, the acquisition of basic numerical skills appears to be relevant to later life perspectives. A meta-analysis by Duncan et al. (2007) even revealed that basic numerical skills are more predictive for later academic achievement as compared to reading and attention skills. As such, exploring ways to stimulate the development of numerical skills seems to be highly relevant on the individual as well as societal level.

According to the *integrated theory of numerical development* (Siegler, 2016), the acquisition of increasingly precise numerical magnitude understanding is the common core of numerical development affecting later arithmetic as well as more advanced mathematics (see also Siegler et al., 2011). Between three and five years of age, children are assumed to develop an understanding of numerical magnitude for numbers up to 10 (Siegler, 2016). Like other theoretical approaches (see e.g., Resnick, 1983; Dehaene, 1992; Fritz et al., 2013), the integrated theory proposes that numerical magnitude is represented spatially along a mental number line. In this context, it has also been proposed that the spatial representation of numerical magnitude is defined with respect to a body-centred egocentric reference frame, where different positions on the mental number line are identified with respect to one’s own body midsagittal plane (e.g., small numbers toward the left, Conson et al., 2009). Moreover, it is assumed that mastery of spatial language terms may help children better grasp spatial aspects of numerical representations, such as spatial relations between numerical magnitudes on a mental number line (see Georges et al., 2021). In the present study, we evaluated the association between preschool-aged children’s knowledge of relations between numerical magnitudes (so-called relative numerical magnitude understanding, see Jirout and Newcombe, 2018) and their use of egocentric reference frames in spatial language (where spatial relationships are described from the viewer’s perspective).

**FIGURE 1:**
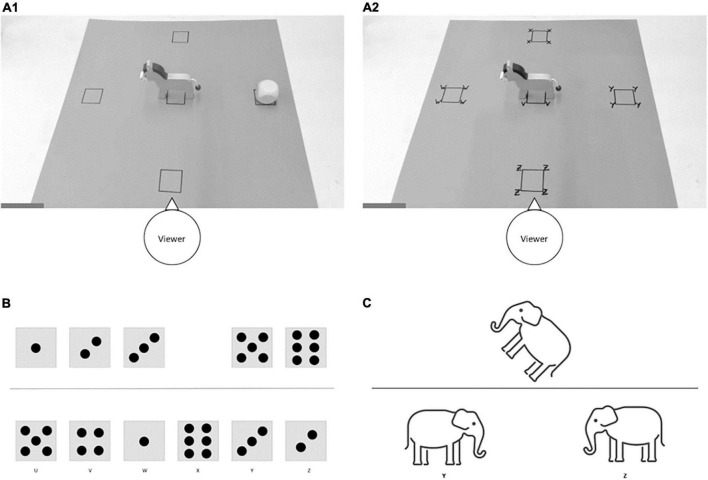
Stimulus material of tasks used. **(A1)** Set up to assess children’s production of spatial terms. Children were asked, “Where is the cube?” An egocentric answer would be “to the right,” while an allocentric answer would be “behind” (the donkey). **(A2)** Set up to assess children’s comprehension of spatial terms. The experimenter told the children where to put the cube and asked them to point to the given position. **(B)** Exemplary stimulus of the task to identify missing dice patterns of a series. **(C)** Exemplary stimulus of the task to assess children’s mental rotation skills.

The assumption of a spatial representation of number magnitude not only plays an important role in models of numerical development. Instead, it also serves as the basis for explaining numerous empirical findings indicating significant associations between numerical and spatial skills (for findings concerning preschool children, see e.g., Gunderson et al., 2012; Zhang et al., 2014; Zhang and Lin, 2015; Cornu et al., 2017, 2018; Frick, 2019; Lonnemann et al., 2019). For instance, Mix et al. (2016) evaluated associations between different basic numerical and spatial skills in a cross-sectional study involving 854 children from kindergarten, third, and sixth grade. They observed that mental rotation skills were most strongly associated with numerical performance in kindergarten, whereas visuospatial working memory and figure copying skills were most strongly associated with mathematical performance in sixth grade. These findings, thus, point to age-related differences in the relationship between numerical-mathematical and spatial skills. However, in a subsequent study involving another cohort of children these age-related differences could not be replicated (Mix et al., 2017; see also Johnson et al., 2022). On the basis of these and other relevant findings, Mix (2019) concluded that the association between numbers and space seems to be stable across development, but that the way specific numerical and spatial skills are associated over time is far from being understood. In fact, there is not even a consensus on the structure and subcomponents of spatial skills (see e.g., Uttal et al., 2013; Newcombe and Shipley, 2015). It is, however, assumed that mastery of spatial concepts involves the consideration of different spatial frames of reference. A frame of reference is a coordinate system that organizes spatial relations. A common taxonomy divides frames of reference into egocentric and allocentric, this means those from one’s own perspective and those from the perspective of another entity, respectively (see Figures 1A1,A2) (e.g., Klatzky, 1998; see also Shusterman and Li, 2016 for more differentiated taxonomies). In this context, Frick (2019) investigated different spatial skills of children in preschool and their associations with mathematical skills in first and second grade. The author reported evidence for two subcomponents of spatial skills, namely for egocentric transformations (i.e., mental rotation and spatial scaling) and for allocentric transformations (e.g., cross-sectioning, perspective taking). Interestingly, egocentric transformation skills were most strongly associated with children’s arithmetic skills. In line with these findings, it has been suggested that egocentric object-based spatial transformations play an important role in mathematical development, while allocentric viewer transformations may not (Gilligan et al., 2019a). Likewise, the representation of different conceptual domains, including number, has recently been proposed to be based on the use of egocentric reference frames derived from sensorimotor experience (Bottini and Doeller, 2020). According to this view, numerical information is processed by neurons in the parietal cortex that represent the location of objects according to an egocentric frame of reference, and this processing is thought to be shaped by sensorimotor experience, as for example suggested by the finding that number processing is modulated by finger-counting habits, such that numbers are more likely to unfold to the right in the mind when people habitually count from one to ten starting with their left hand (Fischer, 2008; see also Pitt and Casasanto, 2020). Numerical magnitude information may thus be spatially represented ascending from left-to-right in an egocentric frame of reference with different positions on a mental number line being identified in relation to one’s own body midsagittal plane (see Conson et al., 2009). Following from this, the present study addresses the question whether the use of egocentric reference frames in spatial information processing is associated with the numerical magnitude understanding of children in preschool age.

Generally, the use of spatial reference frames plays an important role in the development of spatial language, with the latter being crucial for the development of spatial skills (see Wu et al., 2021 for an overview). Spatial language has been described as “a means of representing objects and locations through verbal description with respect to multiple [spatial] coordinate systems or frames of reference” (Binder et al., 2009). Among English-speaking children, it was shown that the majority of four-year-olds was able to indicate the position of a teddy placed in (100%), on (90%), under (75%), and in front of a box (75%), while only a smaller part of them was able to indicate the position of the teddy placed behind (50%), above (10%), below (0%), to the left (40%), and to the right (40%) of the box (Farran and Atkinson, 2016). When asked to place the teddy in different locations, children’s performance was better, but even among seven-year-olds, correct responses of all children were only observed when they were asked to indicate the position of the teddy placed in and on the box, suggesting that children at this age have not yet acquired comprehensive spatial language skills (Farran and Atkinson, 2016). Comprehensive spatial language skills involve the consideration of different spatial reference frames; this means that children need to understand that spatial relationships can be described from the viewer’s perspective (egocentric frame of reference) and from the perspective of a directed ground entity (allocentric frame of reference) (see Figures 1A1,A2). By using a non-directed ground entity (a box), Farran and Atkinson (2016) restricted their spatial language comprehension and production tasks to the viewer’s perspective. In tasks involving consideration of another entity’s perspective, even at the age of 11 years, not all children apply the words right and left properly (e.g., Rigal, 1994).

Spatial language is not only thought to play a crucial role in the development of spatial skills (see Wu et al., 2021 for an overview), but also to be associated with numerical skills. For instance, Purpura and Reid (2016) showed that 3- to 5-year-olds’ numerical skills were associated with their so-called mathematical language skills, which were composed of quantitative (e.g., take away, a little bit, more) and spatial language skills (e.g., nearest, under, first, far). In a similar study, Hornburg et al. (2018) examined associations between 3- to 6-year-old children’s mathematical language and specific basic numerical skills. The authors observed that children’s mathematical language, measured by their quantitative and spatial language skills, was associated significantly with their performance in tasks on verbal counting, one-to-one correspondence, Arabic numeral identification, cardinality understanding, comparisons of sets and/or numerals, ordering numerals, and story problems. A training study even revealed that 3- to 5-year-old children who participated in a dialogic reading intervention that involved talking about pre-determined questions focusing on quantitative and spatial language showed significantly enhanced numerical skills compared to a business-as-usual control group (Purpura et al., 2017). Moreover, in a recent longitudinal study (with a pre- and post-test interval of 6 weeks), Chan et al. (2022) examined associations of 5- to 6-year-old’s relational language (i.e., combining quantitative, spatial as well as temporal contexts such as more-less, top-bottom, and begin-end) and their number relation skills, as assessed by two tasks on cardinal relations among numbers (i.e., number comparison and set relation), one task on ordinal relations (i.e., number ordering), and one task on number-space mapping (i.e., number line estimation). The authors found that relational language skills predicted later number relations skills, in particular for children’s performance in number line estimation and even after controlling for vocabulary, executive functions, but also counting and number identification skills.

Rather than examining children’s mathematical or relational language skills, a few studies focused more specifically on children’s spatial language skills and their potential link to numerical skills. In this context, Verdine et al. (2014) examined the relationship between numerical and spatial skills in 3-year-old children by asking parents to report spatial language terms they use in interaction with their children (e.g., large, between, below, behind). To assess children’s spatial skills, they used a spatial assembly task in which children had to build block constructions from models. Children’s numerical skills were assessed in terms of (i) counting to the highest number children could do without an error, (ii) a give-a-number task requiring children to give a specific number of objects to the experimenter, (iii) naming the successor of given numbers, and (iv) solving non-verbal addition and subtraction tasks using tokens. Results showed that the number of spatial words parents used in their interaction with children was significantly related with children’s spatial as well as numerical skills. Importantly, these associations remained significant even after considering children’s general language skills. In addition, Bower et al. (2020) showed that 3-year-olds’ comprehension of spatial terms (e.g., under, between, in, behind) was associated with their spatial (i.e., reproduction of 2D and 3D models) and numerical skills (i.e., subitizing, number constancy, numeracy, magnitude comparison, and basic addition and subtraction with tokens). Recently, Georges et al. (2021) reported that 4- to 6-year-old’s spatial language skills assessed by the production and comprehension of spatial terms (e.g., on, left, before, above) were associated significantly with their verbal number skills (i.e., forward and backward counting, naming Arabic numerals) even when controlling for influences of verbal and visuospatial skills, age, sex, and SES.

According to Georges et al. (2021), mastery of spatial language terms may help children better grasp spatial aspects of numerical representations, such as spatial relations between numerical magnitudes on a mental number line. In this context, Jirout and Newcombe (2018) proposed to distinguish between absolute and relative numerical magnitude understanding and to focus on relative magnitude understanding when considering associations between numerical and spatial skills. While absolute number magnitude is about answering questions such as “how many?,” relative number magnitude understanding is about answering questions such as “how much/many compared to what?” (Jirout and Newcombe, 2018). Accordingly, the relevance of the mastery of spatial language terms for grasping spatial relations between numerical magnitudes proposed by Georges et al. (2021) should be reflected in a specific association between children’s relative numerical magnitude understanding and their spatial language skills.

In the present study, we sought to evaluate this assumption in preschool-aged children. Based on the notion that the representation of numerical information relies on the use of egocentric reference frames (e.g., Bottini and Doeller, 2020), as well as recent evidence suggesting that egocentric spatial transformation skills play an important role in mathematical development (Frick, 2019; see also Gilligan et al., 2019a), we first hypothesized that the association between children’s relative numerical magnitude understanding and their spatial language skills is specifically detectable for spatial language processing, involving the use of egocentric reference frames. Therefore we assessed children’s spatial language skills in tasks that allow for responses using egocentric and allocentric frames of reference as well as their relative numerical magnitude understanding using a task that does not have an inherent spatial character (in contrast, for example, to the number line estimation task, see Siegler and Opfer, 2003). To show that the hypothesized association cannot be explained by individual differences in absolute numerical magnitude understanding or knowledge on numerical sequences (e.g., knowing that “6 comes before 7”; see Lyons and Beilock, 2009; Colomé and Noël, 2012), the respective skills were also assessed as control variables. As mental rotation processes might play a role in both spatial language processing (see e.g., Shusterman and Li, 2016) and numerical processing (see e.g., Frick, 2019; Gilligan et al., 2019a), these were also measured as control variables. Moreover, children’s SES, their native language, age, and sex were considered as control variables. Second, we hypothesized that the association between children’s use of egocentric reference frames in spatial language and their relative numerical magnitude understanding is stronger than the association between children’s use of allocentric reference frames in spatial language and their relative numerical magnitude understanding. To see how adults compared to children perform in the spatial language task, this task was additionally carried out by adults.

## Materials and methods

### Participants

A total of 60 German-speaking children (30 girls, 30 boys) participated in this online study. Mean age of the children was 4;10 (*M*_age_ = 58.8 months, SD = 4.48). One of the children completed the assessment in two sessions instead of one. Data from two children were not considered for analyses because the sound was not working for one child and for the other child the person present during the session was constantly influencing the child. As not all children completed all tasks or the parents present took influence on their child in individual tasks, the main analysis is based on a sample of 52 children. Children were recruited throughout Germany. The study was approved by the local ethics committee of the *University of Potsdam, Germany* (under application number 55-2020).

Additionally, a total of 25 German-speaking adults participated in this study as comparison group and completed the spatial language task. Three participants were excluded from the analysis because two persons were already familiar with the task and another one was an outlier in terms of age (*z* = 3.81). The final sample included 22 participants (17 women, 5 men; *M*_age_ = 25.14 years, SD = 5.26).

### Procedure

Due to the restrictions caused by the Corona pandemic, children were tested individually in a video call and thus participated remotely. During the session, a parent and an experimenter were present together with the child. The parent was instructed not to help the child in any way, for instance by giving tips or other advice. Participating children and their parents joined the session from their private laptop. The experimenter presented the tasks while talking to the child *via* the camera, split screen, or external webcam. The assessment was performed in one session and lasted about 30 min. Children completed a task to assess their spatial language skills, a mental rotation task, and four numerical tasks. The numerical tasks included (i) a task to assess relative (number words comparison), (ii) absolute numerical magnitude understanding (number word comprehension), and (iii) two tasks to assess knowledge on numerical sequences (identification of predecessors and successors of given numbers as well as identification of missing dice patterns of a series).

Tasks used were presented in the following order: (i) number word comprehension, (ii) identification of predecessors and successors of given numbers, (iii) number words comparison, (iv) identification of missing dice patterns of a series, (v) spatial language, and (vi) mental rotation. There were two versions (A and B) with different item order for all tasks. Each child received one of the two variants (A or B consistently for all tasks used) which was counterbalanced across sex. One child received version A for one part of the tasks (i.e., spatial language) and version B for the other part (i.e., for all numerical tasks and mental rotation) by mistake. For maintaining motivation, the children received a stamp on a certificate after each completed task, which the experimenter showed to the children *via* the camera after each stamp. Before the session, parents were asked to answer questions about their socioeconomic background in an online questionnaire. More details on the respective measures is provided below.

The procedure for the spatial language task with the adults was identical to that with the children. In contrast to the children, adult participants received financial compensation for their participation.

#### Spatial language skills

To evaluate children’s spatial language skills, the production and comprehension of spatial terms was assessed. Both tasks were performed consecutively, always starting with the production of spatial terms in order not to bias any terms. Children were shown a pad with position markers *via* a webcam.

To assess children’s *production of spatial language terms*, the experimenter placed a donkey and a cube in different positions on the pad. The donkey was placed at the central position on the pad (see Figure 1A1). The child and the experimenter always had the same perspective, and from this perspective the donkey always looked to the left. It was taken away after each trial and repositioned for the next trial. The cube was placed to the right of the donkey, behind the donkey, in front of the donkey, and to the left of the donkey (in this order, seen from the donkey’s perspective; Version A), or the cube was placed behind the donkey, to the right of the donkey, to the left of the donkey, and in front of the donkey (in this order, seen from the donkey’s perspective; Version B). The task started with an example in which the donkey was placed so that it was facing the child and the cube was placed under the donkey. In each trial, children were asked: “Where is the cube?”. Children were not given feedback as to the correctness of their answers. In each trial, they could answer either from their own perspective (egocentric) or from the donkey’s perspective (allocentric). For example, in Figure 1A1, an answer from the egocentric perspective would be that the cube is “to the right,” while an answer from the allocentric perspective would be that the cube is “behind” (the donkey). Children were not asked to respond either allocentric or egocentric because prompts such as “from your perspective” or “from the donkey’s perspective” are complex and can be difficult to convey. To rule out the possibility of children’s performance being affected by not understanding such prompts, questions were asked in everyday language and in an open-ended manner. The number of responses that corresponded to an egocentric perspective on the one hand and the number of responses that corresponded to an allocentric perspective on the other hand were used as dependent variables in this task. Due to the fact that most of the children and likewise a part of the adults answered with above and below instead of the terms behind and in front of (from an egocentric perspective), these answers were also considered correct (see Supplementary Table 1).

To assess children’s *comprehension of spatial language terms*, the experimenter told the children where to put the cube and asked them to point to the given position. To determine which position the children pointed to, the pad with the position markers showed a letter (V, W, X, Y, Z) at each marker, and parents were asked to indicate the letter corresponding to their child’s answer to the experimenter. The experimenter then placed the cube in the respective position. Again, the donkey was placed on the middle position marker of the pad (see Figure 1A2), so that the child and the experimenter always had the same perspective with the donkey looking to the left. As in the production task version, the donkey was removed after each trial and repositioned for the next trial.

Children should successively locate the following positions: in front of the donkey, to the left of the donkey, to the right of the donkey, and behind the donkey (in this order, seen from the donkey’s perspective; Version A), or in the following order: to the left, in front of, behind, to the right (in this order, seen from the donkey’s perspective; Version B). The task started with an example in which the donkey was placed so that it was facing the child and children were told that the cube should be under the donkey and asked to point to this location. Children were not given feedback as to the correctness of their answers. In each trial, they could answer either from their own perspective (egocentric) or from the donkey’s perspective (allocentric). The number of responses that corresponded to an egocentric perspective on the one hand and the number of responses that corresponded to an allocentric perspective on the other hand were used as dependent variables in this task.

Production and comprehension of spatial terms were significantly correlated [egocentric: *r*(52) = 0.401 *p* = 0.003; allocentric: *r*(52) = 0.508 *p* < 0.001] and combined into total scores for further analyses (see Georges et al., 2021 for a similar procedure). Internal consistency indicated by Cronbach’s alpha was α = 0.71 (egocentric) and α = 0.76 (allocentric).

#### Number words comparison

In the *number words comparison task*, the experimenter named two numbers aloud and the child was asked to decide which number was more or less and state the respective number. In a first block of six trials, children were asked to decide which number was more and in a second block of six trials, they were asked to indicate which number was less. Children received the pairs of numbers in the order 5–3, 6–8, 7–9, 8–5, 15–17, 20–10, 3–2, 4–6, 5–7, 9–8, 11–12, and 19–18 (Version A), or in the order 3–2, 4–6, 5–7, 9–8, 11–12, 19–18, 5–3, 6–8, 7–9, 8–5, 15–17, and 20–10 (Version B). They were not given feedback as to the correctness of their answers. The number of correctly solved items was used as dependent variable in this task. Internal consistency indicated by Cronbach’s alpha was α = 0.65.

#### Number word comprehension

To assess children’s *number word comprehension*, they were asked to show different quantities using their fingers (e.g., “Can you show me four fingers?”). Children were asked to show 4, 5, 7, 9, 0, 8, 10, and 6 fingers to the experimenter (Version A), or they were asked to show 4, 5, 6, 10, 8, 0, 9, and 7 fingers to the experimenter (Version B). Again, children were not given feedback as to the correctness of their answers. The sum of correctly solved items was used as dependent variable in this task. Internal consistency indicated by Cronbach’s alpha was α = 0.77.

#### Knowledge on numerical sequences

Children’s *knowledge on numerical sequences* was assessed in two ways: they had to (i) identify predecessors and successors of given numbers and (ii) to identify missing dice patterns of a series.

Regarding (i) children were asked to state the number that comes exactly after or before a given number when counting. In the first block of six trials, children were asked to indicate successors and in a second block of six trials, they were asked to name predecessors. The experimenter started each block with an example. Children completed numbers in the order 3, 5, 6, 9, 13, 18, 4, 6, 8, 9, 12, and 17 (Version A), or in the order 4, 6, 8, 9, 12, 17, 3, 5, 6, 9, 13, and 18 (Version B). Children were not given feedback as to the correctness of their answers. The number of correctly solved items was used as dependent variable in this task. Internal consistency indicated by Cronbach’s alpha was α = 0.85.

Regarding (ii) children were presented with pictures, each with five dice patterns arranged horizontally in ascending order from left to right, with a gap in one position (see Figure 1B). Separated by a horizontal line, all six dice patterns were arranged horizontally in random order. Children were asked, which dice pattern belongs in the gap and to point to this dice pattern in the bottom row. To determine which position the children pointed to, the dice patterns in the bottom row were marked with a letter (U, V, W, X, Y, Z), and parents were instructed to tell the experimenter the corresponding letter. Children were asked for the missing dice patterns 4, 1, 3, 6, 2, and 5 (Version A), or for the missing dice patterns 5, 2, 6, 3, 1, and 4 (Version B). They were not given feedback on the correctness of their answers. The sum of correctly solved items was used as dependent variable in this task. Internal consistency indicated by Cronbach’s alpha was α = 0.93.

#### Mental rotation

To assess *mental rotation skills*, we adapted the task used by Gilligan et al. (2019b). On each trial, children had to indicate which of two animal images below a horizontal line matched the target image above the line (see Figure 1C). Two different animals (elephant and horse) were used. Images within an item always contained the same animal. The animals were colored consistently per item in either blue, yellow, green or red. The target image was rotated by a certain angle and the probe images below the line were a version of the target image without rotation and a mirror image of the target image without rotation.

Children were asked to point to the image below the line that matched the image above the line (e.g., “Which one of these two horses here matches that one up there?”). To determine which position the children pointed to, the images below the line were marked with a letter (Y, Z), and parents were instructed to indicate the respective letter to the experimenter. Children completed four practice trials, two with a rotation angle of 0° and two with 22°. These were presented in alternating order starting with 0° and feedback was given to the children. This was followed by 20 experimental trials in which no feedback was given as to the correctness of their answers. Trials were separated by a fixation cross.

The experimental trials included an equal number of clockwise and anticlockwise rotations at 45°, 90°, and 135° (four trials for each angle of rotation; two clockwise and two anticlockwise) and four trials rotated by 180° and 0° each. The experimental trials were divided into two blocks, each including all the different angles of rotation. Both blocks thus comprised ten trials each and were performed consecutively. The order of the trials was pseudorandomized so that no two items with the same rotation angle were presented consecutively. In version A children worked through the experimental trials in pseudorandomized order, while in version B they completed the trials in reverse order. Half of the correct responses were on the right (or left) side below the horizontal line. In addition, animals were facing each other below the horizontal line or were facing away from each other. This was balanced for the correct sides (right or left) per item. The number of correctly solved items was used as an estimate of children’s mental rotation skills. Internal consistency indicated by Cronbach’s alpha was α = 0.67.

#### Socioeconomic status

To assess children’s *socioeconomic status*, parents were asked to complete a survey prior to the assessment including two variables (i) time together and (ii) education.

Regarding (i) parents were asked how often it happens at their home that they or a close family member (e.g., partner, grandparent) spend the weekend (a) sit at the table with their child and have a meal together, (b) explore something with their child, (c) play something together with their child, (d) have extensive time to talk with their child, (e) do something with their child, and (f) look at a book with their child and talk about it. Parents could choose between answers categories never, rarely, often, very often. Points were awarded for each item in ascending order, with one point awarded for the answer never and four points for the answer very often. Item scores were normed to 1 (by dividing each score by 4), and the combined score was calculated by averaging the normed item scores. The highest score children could obtain on the SES time together scale was 1.

Regarding (ii) parents were asked to indicate, for the father and for the mother, the school they graduated from (values 1–5 in ascending order: No school-leaving qualification; lower secondary school leaving certificate; intermediate school-leaving certificate; specialized A-levels; A-levels), the highest level of educational qualifications [values 1–8 in ascending order: No vocational qualification and not in vocational training; still in vocational school/vocational training; completed vocational/school training (apprenticeship); still studying, degree from a technical college/vocational or technical academy; Bachelor’s degree; university degree (e.g., Master’s degree); doctorate (Ph.D.)], the current type of employment [values 1–6 in ascending order: Currently not employed (e.g., housewife/househusband, retired without additional income); currently not employed (looking for a job, doing voluntary work); on maternity leave, parental leave, parental leave without employment; not employed (Students in school or vocational training/apprenticeship/study/retraining/internship); employed (part-time with a weekly work schedule of less than 35 h); employed (full-time with a workweek of 35 h or more)], and the occupational status (values 1–6 in ascending order: Never been employed; laborer; salaried employee; civil servant; academic freelancer; self-employed). When indicating the highest level of schooling and the highest level of education parents could choose also the answer “other.” A total of three participants chose the answer “other.” This value was excluded from the analysis and replaced by a missing value to ensure an ascending scaling within the items. In addition, parents were asked to indicate how many books there are in their household and how many books their child owns (including e-books and library books). The parents were able to select a predefined number of books from six answer probes (values 1–6 in ascending order: books in their household: 0–10, 11–25, 26–100, 101–200, 201–250, more than 250; books of the child: 0–10, 11–25, 26–50, 51–100, 101–200, more than 200). Item scores were normed to 1 (by dividing each score by its respective number), and the combined score was calculated by averaging the normed item scores. The highest score children could obtain on the SES education scale was 1.

#### Native language

To assess children’s *native language*, parents were asked to indicate which language was the children’s first language. They could choose between German, another language or multilingual and specify children’s other language, but it was only considered whether German was the child’s sole first language (coded as 1, *n* = 46) or not (coded as 0, *n* = 14).

### Analyses

Due to the fact that some variables were not normally distributed (see Table 1) we calculated Spearman rank correlation coefficients (similar results were obtained when the Pearson correlation coefficient was calculated.). To confirm our first hypothesis, we report analyses on the association between children’s use of egocentric spatial language and their relative numerical magnitude understanding. Moreover, analyses testing our main assumption of an association between children’s use of egocentric spatial language and relative numerical magnitude understanding were performed controlling for absolute numerical magnitude understanding and knowledge on numerical sequences, respectively, to show that the hypothesized association cannot be explained by individual differences in absolute numerical magnitude understanding or knowledge on numerical sequences. Partial correlation coefficients are given for all associations evaluated, taking into account the influences of the control variables mental rotation skills, SES, native language, age (in months), and sex. To test our second hypothesis, a comparison of listwise, partial correlation coefficients of (i) the correlation between children’s use of egocentric versus (ii) the correlation between children’s use of allocentric reference frames in spatial language and their relative numerical magnitude understanding was performed using cocor, an R package used for comparison of the magnitude of two correlations (Diedenhofen and Musch, 2015).

**TABLE 1:** Descriptive statistics for the variables assessed in children.

Variable	n	M	SD	Theoretical range	Empirical range	Shapiro–Wilk-Test
Spatial language skills (egocentric)	54	2.04	1.82	0–8	0–6	< 0.001
Spatial language skills (allocentric)	54	3.1	1.89	0–8	0–6	< 0.001
Number words comparison	56	9.09	2.29	0–12	4–12	0.002
Number word comprehension	56	6.02	2.08	0–8	1–8	< 0.001
Predecessors and successors	53	7.26	3.33	0–12	0–12	0.015
Dice patterns	57	4.84	2.05	0–6	0–6	< 0.001
Mental rotation skills	48	12.65	3.25	0–20	7–20	0.003
Age (in months)	58	58,76	4.48	–	48–66	0.047
SES time together	58	0.9	0.11	0–1	0.46–1	< 0.001
SES education	54	0.76	0.10	0–1	0.53–0.95	0.787

## Results

### Descriptive statistic

Descriptive statistics in children are shown in Table 1. For the spatial language task, children had a mean number of 2.04 (SD = 1.82) responses that corresponded to an egocentric perspective and 3.1 (SD = 1.89) responses that corresponded to an allocentric perspective. Three children only adopted the egocentric perspective, 16 children only the allocentric perspective, and 35 children alternated between the two perspectives while completing the spatial language tasks (see Supplementary Figure 1). For the adult participants the mean number of responses that corresponded to an egocentric perspective was 4.27 (SD = 2.96) and 3.4 (SD = 2.66) for responses that corresponded to an allocentric perspective. Five adults only adopted the egocentric perspective, four adults only the allocentric perspective, and 13 adults alternated between the two perspectives while completing the spatial language tasks (see Supplementary Figure 2). For both children and adults, it was the case that when the egocentric perspective was considered, below and above were used instead of in front of and behind. Among children, 96.87% of those who gave responses that corresponded to an egocentric perspective answered below/above instead of in front of/behind, and one child answered both below and in front of (incorrect instead of behind). Among adults, 93.75% of those who gave responses that corresponded to an egocentric perspective answered below instead of in front of, 88.89% answered above instead of behind, and 0% answered both in front of/behind and below/above. Percentages of items identified correctly for the spatial language task can be found in Table 2.

**TABLE 2:** Percentage of items identified correctly in the spatial language task for children and adults separated for the egocentric and allocentric perspective.

	Children		Adults	
Items	Egocentric	Allocentric	Egocentric	Allocentric
Behind*	30.56	62.04	52.27	50
In front of*	30.56	64.81	47.73	54.55
To the right	17.59	15.74	52.27	36.36
To the left	23.15	12.04	61.36	27.27

*For the production of spatial terms, when the egocentric perspective was used, the responses above and below were also considered correct responses for the items behind and in front of.

### Correlational analyses

**FIGURE 2:**
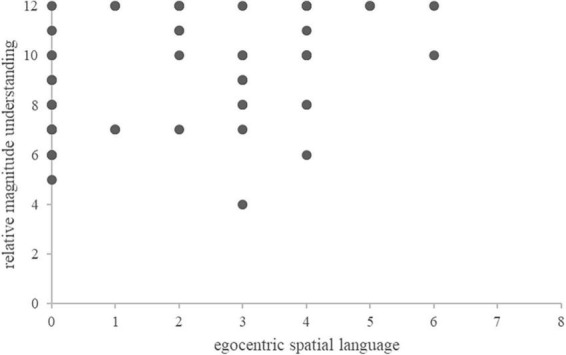
Scatter plot illustrating the correlation between children’s use of egocentric spatial language and their relative magnitude understanding.

The matrix of all bivariate pairwise correlations is shown in Table 3. Partial correlation coefficients are given for all associations evaluated, taking into account the influences of the control variables mental rotation skills, SES, native language, age (in months), and sex. Results confirmed our first hypothesis: With respect to responses that corresponded to an egocentric perspective, children’s spatial language skills were significantly associated with their relative numerical magnitude understanding (i.e., their performance in the number words comparison task), *r*(35) = 0.422, *p* = 0.009 (see Table 3 and Figure 2).

**TABLE 3:** Spearman correlation coefficients for the observed variables (below the diagonal) and partial pairwise spearman correlation coefficients (above the diagonal) taking into account the influences of the control variables mental rotation skills, SES, native language, age, and sex.

	Variable	1	2	3	4	5	6	7	8	9	10	11
1	Spatial language skills (egocentric)	–	–0.489**	0.422**	0.247	0.178	0.149					
2	Spatial language skills (allocentric)	–0.498***	–	–0.088	–0.128	0.081	0.038					
3	Number words comparison	0.394**	0.020	–	0.467**	0.598***	0.463**					
4	Number words comprehension	0.308*	–0.132	0.480***	–	0.631***	0.458**					
5	Predecessors and successors	0.155	0.159	0.626***	0.637***	–	0.555***					
6	Dice patterns	0.253	0.038	0.515***	0.527***	0.556***	–					
7	Mental rotation skills	0.086	0.125	0.223	0.179	0.227	0.277	–				
8	Age (in months)	0.197	0.050	0.254	0.271*	0.317*	0.333*	0.066	–			
9	Sex	–0.083	0.160	0.053	–0.033	0.127	–0.126	0.145	0.070	–		
10	SES time together	0.126	–0.273	–0.186	0.114	–0.066	–0.032	–0.168	–0.019	0.175	–	
11	SES education	–0.155	0.072	0.023	0.123	0.258	0.004	0.057	0.178	0.138	0.327*	–
12	Native language	0.106	–0.134	–0.048	0.066	0.048	–0.002	–0.031	0.239	–0.236	–0.062	–0.135

*p < 0.05. **p < 0.01. ***p < 0.001.

The association of children’s spatial language skills, reflected by responses that corresponded to an egocentric perspective, and their relative numerical magnitude understanding remained significant when accounting separately for influences of children’s absolute numerical magnitude understanding [i.e., their performance in the number word comprehension task, *r*(34) = 0.358, *p* = 0.032], or children’s knowledge on numerical sequences [i.e., their performance in identifying predecessors and successors of given numbers, *r*(34) = 0.400, *p* = 0.016, or their performance in identifying missing dice patterns of a series: *r*(34) = 0.402, *p* = 0.015] as additional control variable. All four reported correlations remained significant after controlling for multiple testing applying the procedure suggested by Holm (1979), where *p*-values are ordered from smallest to largest first and multiplied by their rank to provide the respective adjusted *p*-value.

Results also confirmed our second hypothesis. The comparison of the two correlation coefficients of (i) the correlation between children’s use of egocentric reference frames in spatial language and their relative numerical magnitude understanding, *r*(32) = 0.405, and (ii) the correlation between children’s use of allocentric reference frames in spatial language and their relative numerical magnitude understanding, *r*(32) = -0.283, using Steiger’s test (Steiger, 1980) was significant, *Z* = 2.2917, *p* = 0.022.

## Discussion

The aim of this study was to evaluate the association between preschoolers’ spatial language skills (in terms of their mastery of spatial terms such as in front of, behind, to the left, and to the right) and their relative numerical magnitude understanding. Based on the idea that the representation of numerical information relies on the use of egocentric reference frames (Bottini and Doeller, 2020), as well as recent evidence indicating that egocentric spatial transformation skills play an important role in mathematical development (Frick, 2019; see also Gilligan et al., 2019a), we hypothesized that children’s use of egocentric reference frames in spatial language should be associated with their relative numerical magnitude understanding. In accordance with this hypothesis, results indicated that when considering responses that corresponded to an egocentric perspective, children’s spatial language was significantly associated with their relative numerical magnitude understanding. Furthermore, as hypothesized, the direct comparison showed that responses that corresponded to an egocentric reference frame in spatial language correlated significantly higher with relative numerical magnitude understanding than responses that corresponded to an allocentric reference frame in spatial language. This finding extends our knowledge by pointing to a specific association between preschoolers’ spatial language skills and their relative number magnitude understanding driven by the use of an egocentric reference frame, for the spatial terms in front of, behind, to the left, and to the right.

By using spatial language tasks that allowed for responses that corresponded either to an egocentric or allocentric frame of reference, we were able to show that both the majority of preschool children (64.81%) and the majority of adults (59.09%) gave responses that corresponded to both frames of reference, whereas only a smaller proportion (children: 35.19%, adults: 40.91%) gave responses that corresponded exclusively to one of the two frames of reference. This fits with the results of another recent study showing that children can adopt different frames of reference when acquiring spatial terms (Shusterman and Li, 2016).

As expected, we found that those children who more often gave responses that corresponded to their egocentric perspective in the spatial language tasks also were more proficient at deciding which of two verbally presented numbers is more/less. Based on this, the claim by Georges et al. (2021) on the relevance of the mastery of spatial language terms in grasping spatial relations between numerical magnitudes on a mental number line may be specified as follows: It seems to be the understanding of spatial terms by using an egocentric frame of reference in particular that can be assumed to support relative numerical magnitude understanding. This might reflect that the representation of numerical magnitude relies on the use of an egocentric reference frame, as suggested by Bottini and Doeller (2020). In this context, it has also been proposed that the assumed spatial representation of numerical magnitude in form of a mental number line (e.g., Resnick, 1983; Dehaene, 1992; Fritz et al., 2013; Siegler, 2016) is defined with respect to a body-centred egocentric reference frame, where different positions on the number line are identified with respect to one’s own body midsagittal plane (see Conson et al., 2009).

Based on the present findings, it can further be assumed that mastery of spatial terms may help children not only to process information about sagittal (front, back) and horizontal (to the left, to the right) spatial dimensions, but also to process numerical information on these dimensions in mental space. In other words, mastery of the spatial terms in front of and behind respectively to the left and to the right might enable children to mentally assign numerical magnitudes to a sagittal respectively horizontal spatial dimension and thereby facilitate the comparison of numerical magnitudes. However, it needs to be noted that spatial language skills were assessed online in video calls where no natural three-dimensional depth information was available, and responses in the spatial language production task revealed that both the majority of children and the majority of adults used the spatial terms below (54.39% of all children, and 68.18% of all adults) and above (54.39% of all children, and 72.73% of all adults) instead of in front of and behind. On the basis of these findings, one might therefore assume that it is not only the sagittal and the horizontal, but also the vertical (below, above) dimension to which preschool children may mentally assign numerical magnitudes. Relatedly, findings from a recent study indicated spatial-numerical mapping of similar strength on sagittal, horizontal, as well as vertical axes in five- to nine-year-old children (Cooney et al., 2021, for a review see also Winter et al., 2015).

Importantly, the association observed in the present study between children’s spatial language skills and their relative numerical magnitude understanding cannot be explained by interindividual differences in absolute numerical magnitude understanding or knowledge on numerical sequences, as the association remains significant after considering these former variables as additional control variables. This underscores the assumed relevance of the mastery of spatial language terms in grasping relations between numerical magnitudes (see Georges et al., 2021). Mastery of spatial language terms thus does not seem to be of primary relevance for grasping absolute numerical magnitudes or positions of numbers in the number sequence, at least not in the age group investigated in this study. Instead, its relevance for relative numerical magnitude understanding is also underlined by the findings of a recent study showing that relational language skills are associated with number relation skills (Chan et al., 2022).

The association observed in the present study between children’s spatial language skills and their relative numerical magnitude understanding also cannot be explained by interindividual differences in mental rotation skills, SES, native language, age, and sex. Potentially, mental rotation skills are more strongly related to arithmetic skills (see e.g., Frick, 2019; Gilligan et al., 2019a) and thus may be less able to explain the association between children’s spatial language skills and their relative numerical magnitude understanding. Moreover, mental rotation skills can be assumed to play a dedicated role when left or right is to be evaluated from the perspective of another entity, i.e., an allocentric perspective (see Shusterman and Li, 2016). However, it should be noted that no general language or cognitive skills were measured as control variables in the present study. Associations between spatial language skills and numerical skills have, however, been reported to be significant in previous studies after controlling for general language or cognitive skills (see e.g., Bower et al., 2020; Chan et al., 2022). As such, we chose to assess more specific numerical and spatial control variables. Another limitation of the present study resides in the fact that the sample was relatively small. Additionally, to our knowledge, this is the first study to examine children’s spatial language skills and their association with numerical performance in an online setting. Future studies would therefore be desirable to substantiate the present results also in in-person lab environments. Furthermore, it might be interesting to conduct similar studies with children speaking a language in which geocentrically defined axes (e.g., uphill-downhill as in Tseltal language) are used more prominently than projective, body-defined axes to describe small-scale relationships (see e.g., Abarbanell and Li, 2021). In summary, the present study was the first to examine the association between preschool children’s spatial language and their numerical skills considering spatial frames of reference in spatial language processing. Results extended the current state of knowledge by indicating a specific association between preschoolers’ use of egocentric reference frames in spatial language processing and their relative numerical magnitude understanding. Based on these results, it can be assumed that understanding spatial language terms by using an egocentric frame of reference supports relative numerical magnitude understanding. However, longitudinal and/or training studies are needed to substantiate this claim. When valid, fostering children’s understanding of specific verbal descriptions of spatial positions, for example in the context of parent-child or educator-child interactions, might be a promising way to support children’s numerical development. As suggested by Georges et al. (2021), promoting spatial language in preschool might thus be a successful way to stimulate mathematical development even before the start of formal math instruction.

## Data availability statement

The datasets presented in this study can be found in online repositories. The names of the repository/repositories and accession number(s) can be found below: https://osf.io/hsg8q.

## Ethics statement

The studies involving human participants were reviewed and approved by the Local Ethics Committee of the University of Potsdam, Germany. Written informed consent to participate in this study was provided by the participants’ legal guardian/next of kin.

## Author contributions

NL and JL specified research question, analysis approach, performed the statistical analysis, and drafted the manuscript. All authors designed, set up the study, revised initial draft, and approved the final version of the manuscript.
