# Supplementary material for: Children’s use of egocentric reference frames in spatial language is related to their numerical magnitude understanding
Source: Front Psychol. 2022 Jul 22;13:943191. doi: 10.3389/fpsyg.2022.943191 (PMC9355684; doi:10.3389/fpsyg.2022.943191)
Supplement: Supplementary file 1 [file Data_Sheet_1.pdf]

Supplementary Material

Table S1

Translations of children's and adults' responses from German into English and the responses scored as correct for the spatial language task.

	Responses				Scored as correct			
	Children		Adults		Production		Comprehension	
Items*	Production	Comprehension**	Production	Comprehension**	egocentric	allocentric	egocentric	allocentric
Behind	Behind, To the right/left, Alone, Here, On the side/edge, Next to, In front of	Behind, To the right/left, In front of	Behind, To the right, Next to	Behind, To the right/left	To the right	Behind	To the right	Behind
In front of	In front of, To the left/right, On the (other) side, Next to	In front of, To the right/left, Behind, Under the donkey	In front of, To the left, Next to	In front of, To the left	To the left	In front of	To the left	In front of
To the left	To the right, In front of, Under, Next to, Behind, Alone, On the other side, Under the tree, Straight ahead, Below opposite the donkey	To the left/right, Behind, In front of, Under the donkey	To the left, In front of, Under, Next to	To the left, In front of	In front of, Under	To the left	In front of	To the left
To the right	To the left, Behind, Next to, Above, In front of, On the tree/edge, Straight ahead	To the right/left, In front of, Behind	To the right, Next to, Behind, Above	To the right, Behind	Behind, Above	To the right	Behind	To the right

*Positions from an allocentric perspective; ** Positions chosen by the participants from an allocentric perspective.

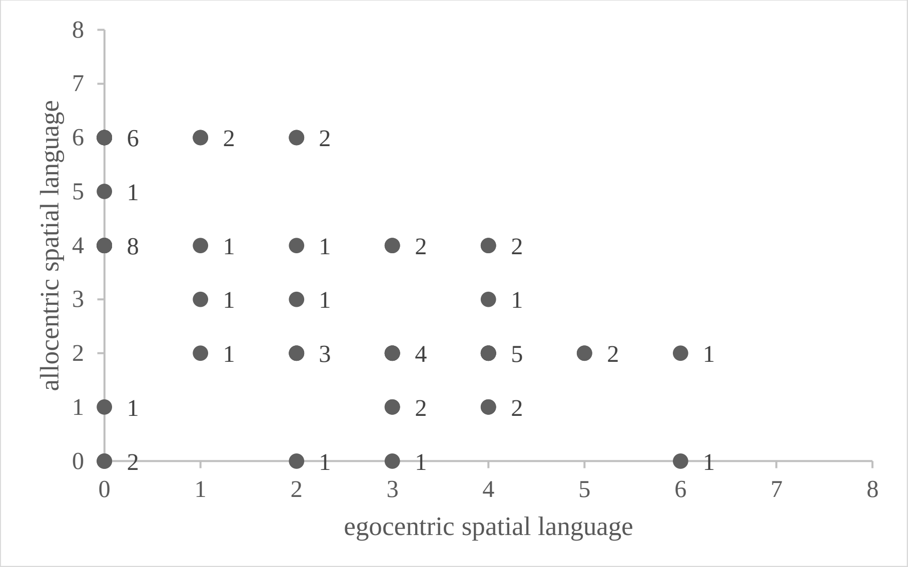

Figure S1. The number of children who correctly adopted the egocentric and/or allocentric perspective. Each dot represents the number of correct responses from the egocentric (x-axis) and from the allocentric (y-axis) perspective and the number next to the dot represents the respective number of children.

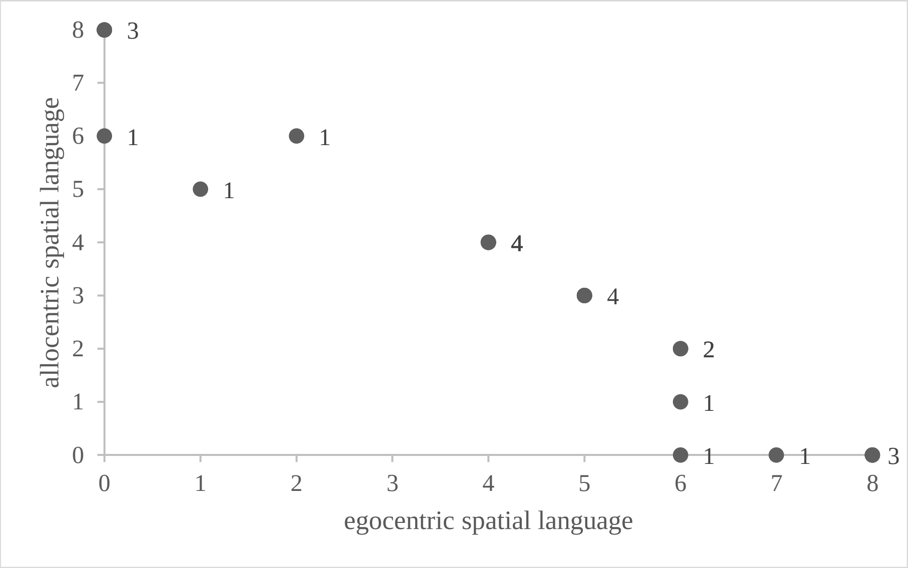

Figure S2. The number of adults who correctly adopted the egocentric and/or allocentric perspective. Each dot represents the number of correct responses from the egocentric (x-axis) and from the allocentric (y-axis) perspective and the number next to the dot represents the respective number of adults.
